# Informed consent for total knee arthroplasty: exploration of patient`s information acquisition and decision-making processes—a qualitative study

**DOI:** 10.1186/s12913-023-09993-5

**Published:** 2023-09-11

**Authors:** Sandro Zacher, Julia Lauberger, Carolin Thiel, Julia Lühnen, Anke Steckelberg

**Affiliations:** 1 Medical Faculty, Institute of Health and Nursing Science, Martin Luther University Halle-Wittenberg, Magdeburger Straße 8, 06112 Halle (Saale), Germany; 2 Medical Faculty, Institute of General Practice and Family Medicine, Martin-Luther-University Halle, Magdeburger Straße 8, 06112 Halle (Saale), Germany

**Keywords:** Total knee arthroplasty, Information needs, Information provision, Decision-making, Informed consent, Patient participation

## Abstract

**Background:**

Total knee arthroplasty (TKA) is an option for the treatment of knee osteoarthritis (OA). Patients have high expectations regarding the benefits of the actual operation. Patients can seek a second opinion on the indication for TKA. In a study, less than half of recommended TKAs were confirmed by the second opinion and conservative treatments are not fully utilized. Informed consent forms that are used in Germany usually do not meet the requirements to support informed decision-making. Our aim was to describe the process from the diagnosis of knee OA through the decision-making process to the informed consent process for TKA, and to understand when, how, and by whom decisions are made. Moreover, we wanted to describe patients' information needs and preferences about knee OA and its treatment, including TKA, and find out what information is provided. We also wanted to find out what information was important for decision-making and identify barriers and facilitators for the optimal use of evidence-based informed consent forms in practice.

**Methods:**

We chose a qualitative approach and conducted semi-structured interviews with patients who were going to receive, have received, or have declined TKA, and with general practitioners (GP), office-based as well as orthopaedists and anaesthesiologists in clinics who obtain informed consent. The interviews were audio-recorded, transcribed and analysed using qualitative content analysis.

**Results:**

We conducted interviews with 13 patients, three GPs, four office-based orthopaedists and seven doctors in clinics who had obtained informed consent. Information needs were modelled on subjective disease theory and information conveyed by the doctors. Patients in this sample predominantly made their decisions without having received sufficient information. Trust in doctors and experiences seemed to be more relevant in this sample than fact-based information. Office-based (GPs, orthopaedists) and orthopaedists in clinics had different understandings of their roles and expectations in terms of providing information.

**Conclusions:**

We were able to identify structural barriers and assumptions that hinder the implementation of evidence-based informed consent forms.

**Supplementary Information:**

The online version contains supplementary material available at 10.1186/s12913-023-09993-5.

## Background

Osteoarthritis is the most common form of arthritis and is associated with pain, loss of function and disability [1]. Total knee arthroplasty (TKA) is an option for the treatment of osteoarthritis (OA) of the knee with the potential for improvements in pain and daily function [2, 3]. TKA is used particularly frequently in Switzerland, the USA, Austria and Germany. With more than 200 surgical treatments per 100,000 persons, these countries have much higher figures than the average of the countries of the Organisation for Economic Co-operation and Development (OECD) (126 per 100,000 population) [2]. In Germany, there are considerable differences in the age-standardised number of knee prostheses at the state level. In 2016, the rate varied between 153 procedures per 100,000 inhabitants and 260 procedures per 100,000 inhabitants [4]. There is no standardised care procedure for the treatment of knee osteoarthritis in Germany. Patients can go first to their general practitioner (GP) with their knee complaint. The GP can refer the patient to an office-based orthopaedic specialist or treat the patient himself or herself. Patients can generally see an office-based orthopaedic specialist without a referral, but some practices expect a referral from the GP. If knee arthroplasty is an option, patients are usually referred to a hospital for a specialist consultation. During this consultation, the indication for surgery is assessed and treatment alternatives are discussed. Some outpatient orthopaedic surgeons have contracts with hospitals where they operate on their patients independently. In this case, the indication for surgery is already made at the office-based orthopaedic specialist. The point at which the consent form is used may vary from hospital to hospital. In the hospital where this study was conducted, the informed consent consultation for TKA with the informed consent form took place after the appointment for TKA had been made. This could be just a few days before surgery or even up to a few weeks before the operation.

Resident doctors are usually involved in the process of informed consent in addition to the surgeons. Since 2015, a minimum number of TKAs is mandatory in Germany. Only hospitals that are expected to perform 50 TKAs per year are allowed to provide the service [5]. Since 2021, patients have a legal right to a second opinion [6]. In a German study on second opinions with 141 participants, only 40% of recommended TKAs were confirmed by a second orthopaedist at an arthroplasty centre. It also showed that conservative treatments, such as physiotherapy or weight reduction, were not implemented consistently beforehand [7]. According to the German consensus-based guideline for the indication of TKA [8], this is a prerequisite, which suggests that the indication is not implemented consistently in clinical practice in Germany. Before the operation, patients have high expectations of the benefits of TKA [9, 10]. There are several studies on satisfaction after the surgery, with heterogeneous results and sometimes considerable limitations regarding the measuring instruments and the quality of the studies [11, 12]. About 20% of patients report unsatisfactory pain reduction [13]. Possible reasons for dissatisfaction could be, for example, unrealistic expectations or a lack of knowledge about possible risks and complications. By providing evidence-based information and by inviting patients to participate in the process of decision making, more patients will be able to make informed choices. Shared decision making is an approach where clinicians and patients make decisions together using the best available evidence and taking patients’ preferences into account [14]. Among others, key aspects of SDM are the transparent presentation of all options as well as an unbiased information transfer to inform realistic expectations regarding possible benefits but also risks and complications [15, 16]. A successful SDM process will lead to informed choices, which comprise the three dimensions knowledge, attitude and behaviour. Choices are informed when adequate knowledge is gained and the attitude is congruent with the behaviour [17]. The process can be supported by evidence-based health information or decision aids through which evidence-based information is provided [14]. Such information is beneficial as it increases the knowledge of options and outcomes and it leads to a more accurate perception of outcome probabilities [18]. If possible, the decision-making process including the provision of evidence based information material should be initiated before the actual consent to the surgery is given. At the latest, however, the information needed to make an informed decision should be provided in the informed consent process. An appropriate informed consent process may – according to the key aspects of SDM – include the presentation of understandable information about the need for and type of surgery, mechanism of action, major risks and consequences, or alternative treatments [19]. The principle of informed consent is based on the human right to self-determination and the ethical principle of autonomy [20, 21], which is also embedded in legal provisions [19, 20, 22]. In Germany, for example, the role of patients was strengthened by the Patient Rights Act in 2013, which was incorporated into the German Civil Code (§§ 630a-630 h BGB). Although verbal consent is sufficient, doctors in Germany often use informed consent forms to support doctor-patient communication and to document written informed consent [23]. These do not usually meet the requirements for supporting informed decision-making [24]. In a current project, we developed evidence-based patient consent forms for surgery and anaesthesia for TKA in order to compare these new consent forms with the standard ones [25]. Since the informed consent to TKA and anaesthesia is the final step in the decision-making process, we wanted to explore the processes of information acquisition and decision-making in order to better understand the context in which the informed consent is imbedded. Our aim was to describe the process from the diagnosis of knee OA through the decision-making process to the informed consent process for TKA, and to understand when, how, and by whom decisions are made. Moreover, we wanted todescribe patients' information needs and preferences about knee OA and its treatment, including TKA, and to find out what information is provided. We also wanted to learn what information is important for decision-making and identify barriers and facilitators for the optimal use of evidence-based informed consent forms in practice.

## Methods

Since the aim of the study is to explore and understand processes and needs, a qualitative approach was chosen. The study protocol was published in the context of the entire project (Sub-study I) [25]. For a comprehensive description, the doctors' perspective was included in addition to the patients' perspective. We followed a descriptive approach. The target sample size was based on the theoretical assumption of data saturation. Therefore, we carried out an iterative process of data collection and data analysis until no new categories emerged and data saturation was assumed. The reporting of the study follows the consolidated criteria for reporting qualitative research (COREQ, Additional file 1) [26].

### Study population

Eligible patients were individuals aged ≥ 18 years with knee OA who had decision-making capacity, understood and spoke German, were considering TKA, declined TKA or had undergone TKA in the past 6 months. In addition, doctors obtaining informed consent (orthopaedists and anaesthesiologists), office-based orthopaedists and GPs were included. We recruited a convenience sample. We contacted office-based doctors in an urban metropolitan area in western Germany by telephone, e-mail or fax. In addition to participation in the qualitative study, patient recruitment was also solicited. Orthopaedists and anaesthesiologists who gained informed consent from patients were recruited through existing contacts with a senior doctor at an academic teaching hospital in a large city in western Germany. The aim was to obtain a broad sample in terms of experience and satisfaction with treatment in general as well as TKA, and including gender and age. We therefore recruited patients through multiple sources. First, recruitment was done via participating office-based doctors using flyers as well as via consultation hours at the participating hospital. Furthermore, recruitment took place via flyers in a rehabilitation facility and via Facebook groups on the topic of knee OA and TKA. People who responded to the flyers and announcements were contacted to check the inclusion criteria. At the beginning of each interview, the participants were informed about the aim of the interview and the background of the interviewer, who was not involved in the treatment. Written informed consent was obtained.

### Data collection

A member of the research team with a nursing background and some experience in interviewing (SZ) conducted semi-structured interviews with patients and doctors. There was no relationship between patients and office-based doctors. A relationship with the doctors in the clinic existed indirectly through the senior doctors involved in the main project. Due to the SARS-CoV-2 pandemic, we conducted telephone interviews. Separate guides were developed for patients and doctors (Additional file 2). Patients were asked to describe their personal course of knee OA, e.g. the initial diagnosis, treatment decisions and the informed consent process, if applicable. We asked the doctors to describe a typical conversation with patients about knee OA or obtaining informed consent. In addition, we asked questions about their interactions with patients. The interview guide was piloted with the first five interviews. No changes were made. We also made field notes on relevant aspects that were mentioned before or after recording. In addition to the demographic data of all participants, which were collected by telephone directly before the interview, supplementary data, e.g. membership in a self-help group, were also collected (Additional file 3).

### Data analysis

The interviews were audio-recorded, transcribed and returned to the participants for checking. We performed a qualitative content analysis according to Mayring [27] using the method of structuring and summarising. Based on the research question, a category system with main and subcategories was deductively created (Additional file 4). Three researchers (SZ, JLa, CT) conducted the analyses. The transcripts were divided among these three persons and each analysed by a second person. After the first interviews, the coding scheme was discussed and adapted for further analyses. During the analysis, the coding scheme could be flexibly adapted and inductively expanded. We used different versions of MAXQDA and Microsoft Excel for the analyses. Within the categories, we looked for differences and similarities among the participants as well as between the patient and the medical perspective, using triangulation. Findings of the analyses were not returned to the participants for feedback. Sociodemographic and supplementary data were analysed descriptively.

## Results

Between November 2020 and July 2021, we conducted interviews with 13 patients, three GPs, four office-based orthopaedists as well as three orthopaedists and four anaesthetists in clinics who were involved in obtaining informed consent. Of the 13 patients, six patients had a TKA in the last six months, six patients planned to have a TKA in the near future and one patient refused the offered TKA. For some patients, the planned or performed TKA was the second, on the other leg. One patient refused arthroplasty of the second knee after the first TKA. With the exception of one anaesthetist, all doctors involved in obtaining informed consent were residents. The participants' characteristics are shown in Table 1.

**Table 1:** Participants' characteristics

		Patients (*n =* 13)		
**Age; mean (range)**		64 (53–77)		
**Sex; n**				
Female		5		
Male		8		
Non-binary		0		
**Graduation; n**				
Secondary school / Vocational training		9		
University degree		4		
**Occupational status; n**				
Full-time employed		4		
Part-time employed		1		
Retired		8		
**Membership in a self-help group; n**		0		
**Years from diagnosis to first surgery/ planned surgery**^**a**^**; mean (range)**		8 (1–17)		
	Office-based orthopaedists, GPs (*n =* 7)	Orthopaedists in clinics (*n =* 3)	Anaesthetists (*n =* 4)	Total (*n =* 14)
**Age; mean (range)**	57 (40–67)	32 (29–36)	33 (29–40)	45 (29–67)
**Sex; n**				
Female	3	1	2	6
Male	4	2	2	8
Non-binary	0	0	0	0
**Medical specialist training; n**	7	0	1	8
**Years of work experience; mean (range)**	27 (4–40)	3 (2–6)	5 (1–10	16 (1–40)
**Contact with patients with knee osteoarthritis on average per week;** **mean, (range)**	13 (3–30)	8 (4–15)	2 (1–3)	9 (1–30)
**Continuing education; n**				
Evidence-based medicine	3	0	0	3
Shared decision-making	1	0	1	2

^a^*n =* 11 (Time of diagnosis unknown; no surgery planned)

**Fig. 1:**
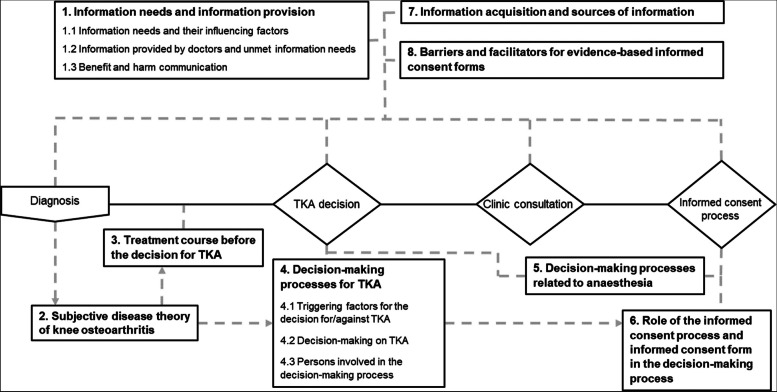
Main categories describing the information and decision-making process from diagnosis to TKA

All the participants completed the interviews. Overall, the interviews lasted on average 29 min (10–46). Interviews with patients lasted on average 36 min (13–46), somewhat longer than interviews with doctors, which lasted on average 22 min (10–33). We assumed data saturation, as the last interviews with patients and doctors did not reveal any new categories. We identified eight main categories describing the information and decision-making process from diagnosis to TKA (Fig. 1) Illustrative quotes are shown in the text and partly in additional tables.

### Information needs and information provision

#### Information needs and their influencing factors

In the process from the diagnosis of osteoarthritis to the informed consent, various information needs were identified (Table 2). The information needs were reported at different time points in the process and in different degrees.

**Table 2:** Information needs of patients in the information process

Disease pattern and treatment	Total knee arthroplasty	Anaesthesia
**Clinical picture of knee osteoarthritis** **Treatment options for knee osteoarthritis** **Treatment / Dealing with limitations in activities of daily living**	**Organisation and technical process** Organisational procedure Operating orthopaedist Surgical procedures Rehabilitation **Complications and risks** General risks and frequency of complications Frequency of failure to improve symptoms after surgery Time until revision surgery is necessary **Course after surgery** Expected restrictions in everyday and leisure activities Expected pain intensity **Prehabilitation**	**Anaesthetic procedures** Possible anaesthetic procedures Sedation under spinal anaesthesia **Complications and risks** General risks and complications Information on individually experienced complications in the past **Course after anaesthesia** Expected restrictions Pain therapy

Patients' needs for information about the clinical picture of knee osteoarthritis and treatment varied. Influencing factors were the extent of the symptoms, the trust placed in the doctor's opinion or recommendation, and whether or not a decision to undergo TKA had already been made.

*“To be quite honest, if the doctor says to me „We have to do such-and-such”, then I don’t need any [more] information.” [009, Patient with planned TKA]*

Patients who had already made a decision before the first consultation in the clinic had a reduced need for information regarding TKA.

*“So a TKA. Yes, and everything else didn’t really matter for me, actually I didn’t need any information because, as I said, I had made my decision.” [015, Patient with TKA]*

The information needs during the informed consent process about TKA and anaesthesia varied. The need for information, including information about complications, was limited by personal preferences and fixed decisions.

*“Yes, they’d explained about the risks. I’ll tackle it this way, quite simply, by every operation something can go wrong. I’ve actually finished with it and filed it away, so to speak. He’d already said a bit about thrombosis etc. But to go into more detail, I don’t give it another thought.” [017, Patient with planned TKA]*

Especially during the informed consent process for anaesthesia, patients with previous negative experiences or fear wanted personalised information for decision-making.

*“Yes, of course you expect to get a lot of information. Personally, you have your own sensitive issues. In my case, it’s a previous anaesthetic where I temporarily lost my short-term memory afterwards. That’s not something you want again, I wanted to avoid that [019, Patient with TKA]*

In contrast, positive previous experience and trust in medical professionals reduced the need for information.

*“Yes, they explained everything. Everything. Some things I didn’t want to know. Where I just say I trust them.” [017, Patient with planned TKA]*

Some patients avoided receiving further information that could lead to uncertainty when they had already made a decision.

*“[…] The more you know, the more uncertain you get. That’s how I see it. Right? Maybe there are a few things you don’t want to hear, where I just say, no, I’ll ask what I would like to know, not what I ought to know.” [017, Patient with planned TKA]*

#### Information provided by doctors and unmet information needs

GPs and office-based orthopaedists offered conservative (shoe inserts, orthoses and bandages, physiotherapy) and surgical (arthroscopy, microfracturing) treatment as well as alternative treatment options (magnet therapy, hyaluronic acid, acupuncture). Information brochures were sometimes used to provide information. In some cases, doctors provided information about the lack of evidence for the treatments. A different perception of their own role in providing information was apparent, as some office-based doctors provided information on TKA and made the indication for TKA, and others left this to the orthopaedists in the hospital. The information provided included both recommendations for surgery and recommendations to postpone surgery as long as possible. The improved quality of life, pain reduction and patient satisfaction were listed as benefits of TKA. When risks were discussed, infection, thrombosis, embolism and the risk of revision were addressed. The information needs of patients in the clinical consultation were largely covered by the information provided. Exceptions were information on prehabilitation and conservative alternatives. Risks were only partly discussed in the clinic consultation and were dealt with in detail only in the informed consent process (Table 3: 1a). Some patients got the impression that the clinic consultation aimed at TKA surgery and was characterised by the recommendation for TKA.

*“I was shocked then. Because I’d thought that something else can happen before the operation. But either the doctor intended to do an operation or they just had time, or whatever, that was my impression, that they wanted to get through it as quickly as possible. And, basically, make me make another appointment right away. Then I didn’t do that. And then this remark came: “We’ll certainly see each other soon.”” [010, Patient who refused TKA]*

**Table 3:** Illustrative quotes for the category “Information needs and information provision”

**1 Information needs and information provision**	
**1.2 Information provided by doctors and unmet information needs**	
1a	*“[The informed consent process] I find it still very important [for decision-making] because beforehand the surgery is usually only outlined so that the patient knows that it’s not a small matter […]. When patients come to me for the informed consent process, most of them are already so resolved that my explanation doesn’t change the opinion any more, nevertheless I have experienced one or two who say Oh, it’s a lot more [Risks] than I thought […]. “ [022, orthopaedist in hospital]*
**1.3 Benefit and harm communication**	
1b	*“Whereby he [explained] the disadvantages quite clearly or that I shouldn’t expect too much. It is optimal if it is fitted correctly and one is pain-free but still has limitations.” [020, Patient with planned TKA]* *“And the fact too that the function of an artificial knee joint cannot be as good as an original joint. That it must also retain certain functional deficits and first and foremost a knee prosthesis is done because of the pain situation and to improve the quality of life. But that in the end functional deficits will remain.” [005, office-based orthopaedist]*
1c	*“But ultimately one has also to explain to the patient that luckily that very rarely occurs and that simply every intervention has complications and that there just has to be a legal safeguard for us somewhere.” [022, orthopaedist in hospital]*
1d	*“I’m just saying that it happens relatively frequently or that it happens very, very seldomly or seldomly. Well, I don’t express it in figures or try to demonstrate it even more [013, Anaesthetist]* *“I also explain about the risk of infection, that this is very seldom and here it is in the region between 2 and at the most 5 percent, so that they feel a bit more, well, just a little bit safer.” [022, orthopaedist in hospital]* *“And then I set it in relation to everyday life, road traffic, household, what can happen to you there, so they then have an idea what it means because I know now that a hip replacement carries a risk of 1:350,000 of older people getting a spinal hematoma. But the risk of an accident on the road in everyday life is greater, then they have an idea and are able to place it. […].” [024, Anaesthetist]*
1e	*“[…] If you can illustrate it somehow, you could say that happens here once in ten thousand times and this is a picture where you can see how much one in ten thousand is. Then, I think, it would certainly be reassuring for a few of the patients.” [013, Anaesthetist]*

Patients reported that all information needs were met during the informed consent process. Information about anaesthesia is not usually provided before the informed consent process.

#### Benefit and harm communication

The way in which benefits and harms are communicated is important in addition to the content of the information provided in the decision-making process. The communication as well as the subjective perception of the benefits of TKA were described both more realistically, with possible limitations left behind, but also with a greater emphasis on the benefits (Table 3: 1b).

Patients generally described an emphasis on the low risks of the operation in the consultations.

*“[In the informed consent process risks] were mentioned, but not particularly emphasized, right? So the emphasis was clearly to state [it is] to over 95 percent completely low-risk.” [006, Patient with TKA]*

This was also expressed in the hospital orthopaedists’ statements (Table 3: 1c).

Doctors communicated risks with verbal descriptors such as very rarely, or with qualitative descriptors and low percentages. One anaesthetist selectively provided natural frequencies in relation to everyday activities (Table 3: 1d). Doctors described challenges in risk communication because the patients do not concern themselves with the probabilities of risks. Furthermore, they saw the need to communicate the risks as being low due to possible patient anxiety.

*“And if patients have really not addressed it at all, then, of course things such as infections will be addressed, how high the chances are, general risks like a pulmonary embolism that can involve life-threatening complications that that/yes, some patients are then likely to be, how shall I put it, “shocked” about everything that might happen. Yes. I try to put it in the correct perspective, what complications are frequent and what are very rare.” [018, orthopaedist in the hospital]*

Doctors did not communicate frequency information since, in their experience, only a few patients wanted this information.

*“Only very few patients really want numerical frequencies. Because that is for many not very easy to place, I think. But if I use words like one in a hundred patients, one in a thousand people, just something simple, that is relatively easy for people to visualize.” [014, Anaesthetist]*

The visualisation of risks was perceived as an opportunity for risk communication (Table 3: 1e).

### Subjective disease theory of knee osteoarthritis

Patients perceived knee OA as an irreparable condition that must be endured and treated with a TKA as an unavoidable procedure and logical consequence.

*“[It was stated that it was an] incipient arthrosis. And I’ll have to live with it for the time being.” [010, Patient who refused TKA]*

This was also supported by the information provided by the doctors (Table 4: 2a). The subjective disease theory influenced decisions in the treatment course. The lack of alternatives anchored in the disease theory or the lack of alternatives to TKA communicated by doctors could be the trigger for the (early) decision to undergo TKA.

*“But [the office-based orthopaedist] said too, there is no alternative for me, didn’t he? Well, now in fear that I belong to these 20 percent where there are still problems, perhaps of a different type or a different pain, he said one way or another I must get a new knee sometime. Right? And it remained for me to think about it, now already or wait another five years?” [011, Patient with planned TKA]*

**Table 4:** Illustrative quotes for the category “Subjective disease theory of knee osteoarthritis”

**2. Subjective disease theory of knee osteoarthritis**	
2a	*“The information I was given that it is, for example, not reparable. It can’t be reversed. That’s the way it is. And it will stay like that, won’t it?” [015, Patient with TKA]*
2b	*“Actually, I had expected to be offered other alternatives. But, really, it was only about operation or no operation? That shocked me a little. And then I said: “No, not so fast.” First, a second opinion, so I went to my family doctor. [010, Patient who refused TKA]*

Nevertheless, this was partly the trigger to get a second opinion or to look for alternatives on their own. (Table 4: 2b).

### Treatment course before the decision for TKA

We identified different treatment courses. We could distinguish between patients who ignored osteoarthritis, who waited and did not undergo any (structured) treatment, or who actively decided to undergo treatment.

*“[Physiotherapy] was offered, wasn’t it? And, as I said, I tried to ignore it and carried on with my daily work […]. Well, really I blocked it out for myself.” [017, Patient with planned TKA]*

For patients who decided to undergo treatment, we could distinguish types who tried several treatments and treatment approaches before deciding to undergo TKA or types who underwent only a few or just one treatment.

*“But as I said, the [magnetic field] didn’t help much, I found […]. Yes, and then I put the whole matter, yes how shall I put it, I put it ad acta for the time being and just carried on like before.” [015, Patient with TKA]*

*“I had the bandage and the shoe inserts and tried it with that […]. Then physiotherapy came in addition. And then [I still have it] I tried microfracturing.” [020, Patient with planned TKA]*

Overall, patients followed conservative, surgical or alternative treatment approaches. The performed treatments could be classified into passive treatments that require no or little behavioural change (e.g. hyalurone injections, magnetic therapy) or more active treatments requiring behavioural change (e.g. physiotherapy).

### Decision-making processes for TKA

#### Triggering factors for the decision for/against TKA

Patients described decisive factors for or against a TKA. The doctor-patient relationship was described as an important factor. The decision was influenced by the perceived competence and recommendation of doctors as well as the perceived trust (Table 5: 4a). Furthermore, patients described their own and others' experiences, for example from their first TKA or from friends, relatives and internet forums, as relevant factors (Table 5: 4b). Disease-related factors, such as the extent of everyday limitations, quality of life, symptoms and the perceived level of suffering, were crucial when opting for or against a TKA (Table 5: 4c). Patients described the corona situation as a reason to postpone surgery (Table 5: 4d). Furthermore, they made their decision for or against TKA dependent on the success of previous treatments. If this has not been effective, then a decision might be made in favour of TKA (Table 5: 4e). In addition, the information provided and the (positive) expectations conveyed were crucial. The expected absence of pain, the improvement in quality of life and unrestricted everyday functions were important factors (Table 5: 4f). Patients also described age and the associated durability of the prosthesis as a reason to postpone TKA (Table 5: 4 g).

**Table 5:** Illustrative quotes for the category “Decision-making processes for TKA”

**4. Decision-making processes for TKA**	
**4.1 Triggering factors for the decision for/against TKA**	
4a	*“And I must say, especially there in the clinic, these doctors, they seem so superior […], keep suggesting alternatives and radiate such confidence that you can’t do anything else.” [009, Patient with planned TKA]* *“Oh, the decision was actually made when he said to me I should definitely get my knee operated. That was the final push. […]“ [025, Patient with TKA]*
4b	*“And then I thought about it and had already read a lot in Internet about how satisfied, let’s say, nearly all of them were with a new knee. […] And now I’ve thought again if I get the chance, the knee could last till the end of life, why should I still wait and keep on dragging myself around here?”* [011, *Patient with planned TKA*]
4c	*“[That was, let’s say] two years before the operation now. Because the thought that that this would happen to me was already clearly present. Only, at that time, the psychological strain was not so great so then I said: “Okay, with the information I have, I’ll wait a bit.” [006, Patient with TKA]* *“Well, for me it’s now an issue where I simply say, I can’t go on any longer. After that, I just got this information from the clinic, so to speak […]” [017, Patient with planned TKA]*
4d	*“At the moment, what with the Corona situation and everything, I don’t think about anything like that.” [010, Patient who refused TKA]*
4e	*“And now it was 50:50 whether I have an arthroscopy or a new knee directly. Then I decided on the arthroscopy because last time that worked quite well.” [025, Patient with TKA]* *“I: Did you have a talk with the orthopaedist who gave you the diagnosis, about the operation again? B: No, I/ we had agreed that if the magnet thing didn’t work, I’d go [to the clinic consultation for TKA].” [009, Patient with planned TKA]*
4f	*“[From the knee arthroplasty I expect] full mobility again […] and of course pain-free.” [025, Patient with TKA]* *“Yes. That I’d be able to do everything I could do before. But that the healing process, as they say, is part of it […] Yes, that I could lead an adequate life, possibly without painkillers. We’ll have to see […]” [017, Patient with planned TKA]*
4 g	*“And then he added that, in theory, it’s quite possible that I would be able to keep this knee joint up to the end, if everything goes normally. And then that was the last reason, where I say, yes, there’s now no other reason to wait.” [011, Patient with planned TKA]*
**4.2 Decision-making on TKA**	
4 h	*“The doctor looked at the documents, looked at it all. No, we’re not doing an arthroscopy, you can come tomorrow, then you’ll get a new knee. Yes, and that was where I just thought, no, not so fast now. It was more like a sort of battering-ram method and then I thought, no, I don’t want that. In the praxis where I was afterwards […] the doctor took much more time.” [025, Patient with TKA]*
4i	*“I hope that my influence is not quite unimportant in the decision-making about when a knee operation should or should not be performed. […] I try to explain what I consider is the right way for the patient, and hope he follows this.” [004, Office-based orthopaedist]*
4j	*“[How such an operation is performed] I didn’t actually know. That’s right. Well, I didn’t look for information about how such a knee operation is performed.” [015, Patient with TKA]*
**4.3 Persons involved in the decision-making process**	
4 k	*“Well, basically, when such crucial or serious decisions are coming up, we do sometimes support each other. In the time before Corona, there was no question about this.” [020, Patient with planned TKA]*
4 k	*“Well, of course, I talked about it with my wife but in the end, it was my decision, wasn’t it?” [006, Patient with TKA]*

#### Decision-making on TKA

Overall, decisions did not seem to be collaboratively structured and the patients' desire to be involved seemed to be low. We were able to describe three ways in which decisions were made:

1. Patients made decisions on their own and only communicated their decision to the orthopaedists.

*“I went to my orthopaedist and told him it’s all getting very strenuous and very difficult. I think it is now time for me to do something. […] And now that’s been confirmed to me here in hospital. […]” [019, Patient with TKA]*

2. Patients trusted and followed doctors' recommendations.

*“Oh, the decision was actually made when he said to me I ought to have a knee operation in any case. That was the final push because otherwise I would get even more problems with my back and, as I said, that was the final push.” [025, Patient with TKA]*

3. Patients expected the doctors to take the initiative and recommend the surgery, and to reinforce their independently made decision.

*“Well, when I went there and said: „I’m in pain”, I was given a prescription for a packet of 100 painkillers. “Do you want a prescription for your knee for massage or something?”, and so on. But in the end, I didn’t feel quite good about it because I thought I need someone who says quite clearly: “Come on, there’s no point any longer, it’s got to go under the knife.” [012, Patient with planned TKA]*

If patients were uncertain about the decision and the clinic consultation was more likely to recommend a TKA, this led to a second opinion or an independent search for alternatives (Table 5: 4 h). Patients described the importance of the doctor-patient discussion as high or low according to their own type of decision-making. Doctors assumed that they involve patients in the decision-making process. They consistently described their own skills in involving patients as good. In the interviews, approaches to informed shared decision-making were partly identifiable.

*“Well, I do try to take care that I don’t tell every patient: “Yes, you’ll benefit from a prosthesis”, but I really say that, yes, there are things that indicate a contradiction, for example. For instance, the patient is young, or the conservative treatment methods have not been exhausted. Yes, I always try in any case to talk to the patient about such matters. […]” [018, orthopaedist in the hospital]*

However, individual statements on the "inclusion" of patients suggest a different understanding of informed shared decision-making (Table 5: 4i).

*„Well, of course always according to what I think, how sensible and how important it is to get a TKA done. […] Some people also have to be pushed towards their good fortune, so to speak.” [003, Office-based orthopaedist]*

The importance of information seemed to be low. In some cases, patients decided to undergo a TKA without any information on TKA (Table 5: 4j).

#### Persons involved in the decision-making process

Other people's experiences were an important source of information for patients. The degree of involvement of friends and relatives in the decision-making process varied. On the one hand, they were actively involved in the process (Table 5: 4 k). On the other hand, TKA was a subject of conversation, but the decision was made alone (Table 5: 4 l). However, some patients also avoided being influenced by the experiences of others in order to be able to make the decision as independently as possible.

*“Well, it was my decision, obviously, you hear from your acquaintances: „He’s got new knees, has a new hip”, or something. But I didn’t let myself be influenced by that, I just say every person is different in their anatomy. Something can always come up. And my decision is definite […].” [017, Patient with planned TKA]*

### Decision-making processes related to anaesthesia

Patients reported that they already made decisions before the informed consent process. The importance of information was reduced, and the decision was made on the basis of their own and others’ previous experiences (Table 6: 5a). Patients described fear of reported or experienced risks as crucial for the decision (Table 6: 5b).

**Table 6:** Illustrative quotes for the category “Decision-making processes related to anaesthesia”

**5. Decision-making processes related to anaesthesia**	
5a	*“I didn’t want spinal anaesthesia. Because I had it years ago when I broke my foot. And I was stupid enough to let them give me spinal anaesthesia. And it was very unpleasant for me. I had that at the back of my head and then I didn’t want that. I knew a bit about general anaesthesia. […] So it was actually clear that I wanted a general anaesthetic, wasn’t it? [–-] My decision was final.” [015, Patient with TKA]*
5b	*„I was afraid [about a spinal anaesthetic]. […] I know of cases where there were complications. A lumbar puncture was done on my daughter and everything happened that I didn’t want to happen. Our neighbour had permanent damage from it when she was young and, well, I simply have these experiences. And that’s why I thought, if I can rule that out, I can put a big tick on it, which comforts me.” [020, Patient with planned TKA]*

### Role of the informed consent process and informed consent form in the decision-making process

The interviews revealed that patients' decisions regarding TKA and anaesthesia were not influenced by the informed consent process. The perceived importance of the informed consent process differed between patients and doctors. Patients rated its importance for the decision about TKA and anaesthesia as low, because the decisions had already been made before.

*“The decision was final. Nothing changed about that [due to the informed consent process].” [006, Patient with TKA]*

However, doctors considered the informed consent process to be important in order to communicate the advantages and disadvantages as well as risks.

*“I find that goes a little in the direction that the informed consent process also has significance for the decision.” [018, orthopaedist in the hospital]*

Patients and doctors perceived the informed consent form as a preparation and aid for the informed consent process. In some cases, patients considered the consent form to be a required document without greater relevance.

*“I just run through the informed consent form, don’t I? The risks are quasi on it. So I use it as a guideline.” [013, Anaesthetist]*

*“Yes. You look through it, you see the pictures about what will roughly be done and so on, you read it through. But that is really just a documentation in my mind, isn’t it? Well/ You inform yourself, but as I said, in hindsight it has to be done anyway.” [015, Patient with TKA]*

### Information acquisition and sources of information

We identified systematic information acquisition strategies, where information was systematically sought, and unsystematic strategies, where information was occasionally found. The independent search for information had a different importance.

*“No, I didn’t inform myself beforehand. Not at all. I let it all, shall we say, pass over me. As I said, if you have confidence in someone, I think, and he knows what he’s doing, then I don’t need any further information.” [015, Patient with TKA]*

*“I’m just a person who hungers after knowledge. […] If I’ve got something [wrong with me], I’d like to know exactly what’s going on and search on the internet or of course I ask the doctors about it. And yes, I just simply want to know what’s going on.” [025, Patient with TKA]*

General sources of information were print and online media, company brochures, TV reports, discussions with doctors, second opinions, personal and third-party experiences, online forums (Facebook) and hospital information events. Patients sought information in addition to consultations with doctors and in the case of insufficient information.

*“[…] The […] doctors’ statements were partly: “We can’t do anything apart from an operation.” So I tried myself, not to cure it, but tried to get information and to find a solution.” [010, Patient who refused TKA]*

### Barriers and facilitators for evidence-based informed consent forms

Based on the interviews, we were able to describe barriers and facilitating factors for the use of an evidence-based informed consent form. Table 7 illustrates both the patients' perspective and the doctors' perspective.

**Table 7:** Barriers and facilitators for evidence-based informed consent forms

Barriers	Facilitating factors
• Decisions were made before the informed consent process without having the information contained in the consent form • Doctors assume that patients are afraid of the frequency of complications, or that it is not relevant for the patients • Patients have a reduced need for information	• Hospital doctors request quantitative visualisations for risk communication • Hospital doctors want better informed patients

## Discussion

In the present study, we were able to describe the information and decision-making processes regarding TKA from diagnosis to surgery by combining the patient's perspective and the doctor's perspective. A main finding regarding the phase of diagnosis and outpatient care was the influence of the subjective disease theory of knee osteoarthritis and the doctor's provision of information. These appeared to be able to substantially model information needs, treatment approaches, and decision-making. Information provided by office-based doctors on treatment options in this sample is not consistently in accordance with the consensus-based guideline for knee OA [28]. For example, treatments without sufficient evidence were offered (e.g. magnetic therapy), or conservative treatment options recommended in the guideline were not offered as described in the literature [29]. The information needs and triggers for decision-making identified in our study are very similar to those found in the literature [30, 31].

As a main result related to the transition to clinical care, it seems that office-based doctors and doctors in hospitals have different understandings of their roles with regard to the provision of information. Orthopaedists in the clinics expected patients to already have some prior knowledge of the benefits and harms of TKA, whereas office-based doctors largely left this information transfer to the clinic. Furthermore, we found that information about benefits was clearly emphasized but risks were only discussed in detail during the informed consent interview. The patient interviews suggest that the general recommendations of the German consensus-based guideline on knee OA regarding the indication for TKA [8] are not consistently followed in practice, especially with regard to the formally required utilization of conservative treatments. This assumption is also confirmed in a study on second opinions [7].

With regard to decision-making, fact-based information might play a subordinate role in contrast to trust in doctors and testimonials. The role of doctors and reports of experiences from friends and acquaintances can also be found in the literature [30, 32]. Decisions were made before the clinical indication or shortly thereafter and thus with limited information. Risks were only discussed in detail during the informed consent interview and were mainly not communicated using the criteria for risk communication [24]. Anaesthesia did not appear to play any role in the decision-making process for TKA. Information regarding anaesthesia was not conveyed until the decision to undergo TKA had already been made. The perception of anaesthesia as a necessary component without including it in the decision for operation is also reflected in the literature [33]. It is obvious that informed shared decision-making is not possible under these conditions. To a large extent, patients in our study also did not seem to expect to be involved in the decision. This contrasts with the study by Suarez-Almazor et al. [32] in which a large proportion wanted to participate.

For the implementation of informed consent forms, as planned in the main study, it can be a challenge that patients seem to have already made a decision before receiving the forms and therefore in some cases avoid further information, or that expert and experience-based knowledge seem to have a higher value than fact-based information. The acceptance of informed consent forms among doctors could be influenced due to the assumption that patients do not wish to receive information about frequencies on risks. Evidence-based information from the onset of diagnosis and doctors trained in evidence-based medicine and informed shared decision-making appear to be important components in enabling participation by patients with knee OA. Further research is needed concerning the importance of the subjective disease theory for the decision-making process. It would be useful to investigate whether providing evidence-based information can influence the subjective disease theory and thus enable an informed decision regarding TKA.

Like the findings of other qualitative studies, the results of the present study may not be generalisable to other contexts. The interviews were conducted in Germany and are thus closely related to the German healthcare system with its unique organizational structures and characteristics. We ensured credibility through comprehensive familiarisation with the material and systematic analysis using the structured analysis steps according to Mayring. Furthermore, a detailed discussion and review of the analysis took place with three researchers and the data of patients and doctors were triangulated. We enabled transferability of our results by describing the German healthcare system for knee OA care and by presenting the categories in detail with a large number of quotes from a wide range of participants. Another strength of the study is the description of the entire process from the diagnosis of knee OA, through the decision for or against TKA, to the informed consent. The study has, however, several limitations. The subjects were a convenience sample and a large proportion of the patients were recruited in the same hospital. Another limitation may have resulted from the restriction of elective surgery during the SARS-CoV-2 pandemic and may have led to selection bias. Credibility may be limited by the lack of participant review of the final categories. Furthermore, although we took field notes, wrote notes and made verbal agreements during the analysis, a reflective journal and detailed description of analytical decisions are not available, which affects dependability and confirmability.

## Conclusion

We were able to identify structural barriers and influencing assumptions of doctors and patients that may have an impact on the treatment process of knee osteoarthritis. The described subjective disease theory with the assumption of the TKA's inevitability can be a trigger for the decision to undergo TKA. In this study, detailed information about the risks of TKA was only provided during the informed consent process, after the decision had already been made and without the risks being communicated in an understandable way. This could also be influenced by the described different role perceptions regarding information transfer between office-based and orthopaedists in the hospital. The assumption by doctors that patients do not want information about frequencies could also have an influence. In addition, the guidelines for the indication of TKA, especially with regard to the utilisation of conservative treatment options, do not seem to have been consistently implemented in this sample. In addition, patients' trust in expert and experiential knowledge, as well as our observation that doctors recommend treatments with insufficient evidence, seem to have an influence on the decision-making process. Overall, these factors appear to have a significant impact on the implementation of evidence-based informed consent forms and generally hinder informed decision-making. Adherence to treatment and indication guidelines should be given higher priority. To promote informed decision-making, the provision of evidence-based information could already be implemented at the diagnosis of knee OA. Furthermore, a reorientation of the informed consent form from a legal safeguard to a supporting tool from the beginning of the decision-making process seems to be a way to promote informed decision-making.

### Supplementary Information

**Additional file 1. **COREQ Checklist.

**Additional file 2. **Interview guides.

**Additional file 3. **Socio-demographics questionnaire.

**Additional file 4. **Category scheme.

## Data Availability

The datasets used and/or analysed during the current study are available from the corresponding author on reasonable request.

## Abbreviations

GP: General practitioners
OA: Osteoarthritis
TKA: Total knee arthroplasty
