# Supplementary material for: Informed consent for total knee arthroplasty: exploration of patient`s information acquisition and decision-making processes—a qualitative study
Source: BMC Health Serv Res. 2023 Sep 11;23:978. doi: 10.1186/s12913-023-09993-5 (PMC10494361; doi:10.1186/s12913-023-09993-5)
Supplement: Supplementary file 1 — Additional file 1. COREQ Checklist. [file 12913_2023_9993_MOESM1_ESM.pdf]

Additional file 1: COREQ Checklist

Tong, A., Sainsbury, P., & Craig, J. (2007). Consolidated criteria for reporting qualitative research (COREQ): a 32-item checklist for interviews and focus groups. *International journal for quality in health care*, *19*(6), 349-357.

Table 1: COREQ

No. Item	Guide questions/description	Reported on page No.
**Domain 1: Research team and reflexivity**		
Personal Characteristics		
1. Interviewer/facilitator	Which author/s conducted the interview or focus group?	5
2. Credentials	What were the researcher's credentials? *E.g. PhD, MD*	-
3. Occupation	What was their occupation at the time of the study?	1
4. Gender	Was the researcher male or female?	1
5. Experience and training	What experience or training did the researcher have?	5
Relationship with participants		
6. Relationship established	Was a relationship established prior to study commencement?	5
7. Participant knowledge of the interviewer	What did the participants know about the researcher? e*.g. personal goals, reasons for doing the research*	5
8. Interviewer characteristics	What characteristics were reported about the interviewer/facilitator? e.g. *Bias, assumptions, reasons and interests in the research topic*	5

Table 1: continued

No. Item	Guide questions/description	Reported on page No.
**Domain 2: study design**		
Theoretical framework		
9. Methodological orientation and Theory	What methodological orientation was stated to underpin the study? *e.g. grounded theory, discourse analysis, ethnography, phenomenology, content analysis*	4
Participant selection		
10. Sampling	How were participants selected? *e.g. purposive, convenience, consecutive, snowball*	4
11. Method of approach	How were participants approached? e.*g. face-to-face, telephone, mail, email*	4-5
12. Sample size	How many participants were in the study?	6
13. Non-participation	How many people refused to participate or dropped out? Reasons?	6
Setting		
14. Setting of data collection	Where was the data collected? e.*g. home, clinic, workplace*	5
15. Presence of non-participants	Was anyone else present besides the participants and researchers?	5
16. Description of sample	What are the important characteristics of the sample? *e.g. demographic data, date*	6-7
Data collection		
17. Interview guide	Were questions, prompts, guides provided by the authors? Was it pilot tested?	5
18. Repeat interviews	Were repeat interviews carried out? If yes, how many?	5
19. Audio/visual recording	Did the research use audio or visual recording to collect the data?	5

Table 1: continued

No. Item	Guide questions/description	Reported on page No.
20. Field notes	Were field notes made during and/or after the interview or focus group?	5
21. Duration	What was the duration of the interviews or focus group?	7
22. Data saturation	Was data saturation discussed?	7
23. Transcripts returned	Were transcripts returned to participants for comment and/or correction?	5
**Domain 3: analysis and findings**		
Data analysis		
24. Number of data coders	How many data coders coded the data?	5
25. Description of the coding tree	Did authors provide a description of the coding tree?	5
26. Derivation of themes	Were themes identified in advance or derived from the data?	5-6
27. Software	What software, if applicable, was used to manage the data?	6
28. Participant checking	Did participants provide feedback on the findings?	6
Reporting		
29. Quotations presented	Were participant quotations presented to illustrate the themes / findings? Was each quotation identified? e.*g. participant number*	9-20
30. Data and findings consistent	Was there consistency between the data presented and the findings?	9-20
31. Clarity of major themes	Were major themes clearly presented in the findings?	9-20
32. Clarity of minor themes	Is there a description of diverse cases or discussion of minor themes?	9-20
