# Supplementary material for: Informed consent for total knee arthroplasty: exploration of patient`s information acquisition and decision-making processes—a qualitative study
Source: BMC Health Serv Res. 2023 Sep 11;23:978. doi: 10.1186/s12913-023-09993-5 (PMC10494361; doi:10.1186/s12913-023-09993-5)
Supplement: Supplementary file 2 — Additional file 2. Interview guides. [file 12913_2023_9993_MOESM2_ESM.pdf]

Additional file 2: Interview guides

1. Guiding questions for patients

Situation: Diagnosis by general practitioner/specialist

- How did you learn about your knee osteoarthritis?
    - How did the diagnosis proceed? (Referral to specialist?)
    - From which doctor did you receive the diagnosis?
    - Did you already have a suspicion before? Had you already searched for information?
- How long ago was the diagnosis made? Can you still describe the course of the conversation?
    - What expectations did you have of the interview?
        - Were they fulfilled? What else would you have liked to learn?
- Describe the information you received about the course and treatment of knee osteoarthritis.
    - How important was this information for you?
    - Did you receive any information material? (Type: electronic, video, analogue)
    - Did you miss any information?
    - Did you search for it elsewhere?

Situation: Transition diagnosis/treatment

- What was the next step for you after diagnosis?
    - Did you look for further information?
        - On what topics did you want information?
    - Where did you find this information?
        - Internet, friends, relatives, professionals?
        - How would you rate the information you found?
    - How would you describe the importance of information from relatives and friends/acquaintances?
    - Describe any overlapping or differences in the information from your doctor.
- What was the process of deciding on treatment?
    - How would you describe your involvement in the decision?
    - Describe how your questions were handled.
    - What value did the decision have for you? (e.g., investing time)

- How would you describe your satisfaction with the decision-making process?
    - Why were you satisfied/why were you not satisfied?
- What treatment options were suggested to you? (or: can you remember what treatment options were suggested)?
- Describe the treatment(s) you received.
    - How did the decision come about?
    - How would you describe your satisfaction with the treatment?
    - What, if any, led to the decision to discontinue treatment?
- Did you receive any information from other healthcare professionals during your treatment? (e.g., physical therapy, alternative practitioner, medical supply store).
    - What information did you receive?
    - What value did this information have for you?
    - Describe any overlapping or differences in the information you received from your doctor.
    - How would you describe your satisfaction with the information you received?
        - Why were you satisfied/why were you not satisfied?

Situation: decision to have surgery

- How did your decision to have the surgery occur?
    - Did you make the decision with someone?
    - What factors played a role in the decision?
- How would you describe the conversation that led to the decision to have surgery?
    - What were your expectations of the conversation?
        - Were they met? What else would you have liked to have learned?
    - How would you describe your involvement in the decision?
    - Describe how your questions were handled.
    - What value did the decision have for you? (e.g., investing time)
    - How would you describe your confidence in the decision?
- How would you describe the information you received from your doctor about the surgery?
    - What information would you like to have had?
    - Have you received any information material?
        - Type (electronic, paper-based, video, ...).
    - Would you have liked more information?

        - If so, which sort of information?
    - Did you have any questions?
        - Which ones? / How would you describe your satisfaction with the answers?
    - Have you already received information about anaesthesia?
        - Would you have liked information on this beforehand?
    - Did you independently search for further information?
        - On what topics did you wish you had received information?
        - Where did you find this information?
        - How would you rate the information you found?
        - Describe any overlappings or differences in the information provided by your doctor.

Situation: Informed consent process

- How would you describe the informed consent process?
    - How would you describe the organizational process?
        - Surgery/Anaesthesia
        - Time of receiving the informed consent form
        - Place
        - Time before surgery
        - Time after decision
        - Total time
        - Was the informing orthopaedist also the one operating?
    - How would you describe the information you received?
        - What is the importance of the informed consent form?
        - What type of information did you receive (electronic, paper, video)?
        - Describe any overlapping or differences in the information you received
    - What information would you like to receive?
        - Was all the information you would like available?
        - What was not available?
- How would you describe the process of the informed consent process?
    - Were personal needs addressed?
        - What additional information would you have liked?
- How would you describe the influence of the informed consent process on your decision?

Situation: After the operation

- Looking back, is there anything you wish you had known more about? –
- Did anything surprise you?
- Did something occur that you were not informed about?

2. Guiding questions for referring doctors and clinicians

a) Referring doctors

- How would you describe a typical conversation about the course and treatment of gonarthrosis?
    - What information do you give to patients?
    - Do you give the patients information material?
        - What/why these?
- Do you feel well prepared to educate the patient about treatment options and their risks as well as their alternatives?
    - What would you like to have in order to feel better prepared?
    - Would you like to have assistive material? (evidence-based information)
- How well do you succeed in involving patients in decision-making?
    - Are there facilitating factors and barriers?
- What is your experience with patients who already have information from other sources?
    - Do you address the information they bring with them? How do you do this?
    - How do you describe the impact on your consultation?
- How would you describe the decision-making process with your patients?

b) Clinicians

- How would you describe a typical informed consent process for TKA?
    - What information do you give to the patients
    - Do you give information material to the patients?
        - Which ones/why these?
- How would you describe the importance of the informed consent process in decision-making?
- Describe the importance of the informed consent form for decision-making.
- How well do you succeed in involving patients in decision-making?

    - Are there facilitating factors and barriers?
- Do you feel well prepared to inform the patient about treatment options and their risks as well as their alternatives?
    - How would you assess the relevance?
    - What would you like to have in order to feel better prepared (e.g. evidence-based information)?
- How well do you succeed in involving patients in decision-making?
    - Are there facilitating factors and barriers?
- In your opinion, which factors are conducive or obstructive to the informed consent process?
- What is your experience with patients who already have information from other sources?
    - Do you address the information they bring with them? How do you do this?
    - How would you describe the impact on your consultation?
