# Supplementary material for: Informed consent for total knee arthroplasty: exploration of patient`s information acquisition and decision-making processes—a qualitative study
Source: BMC Health Serv Res. 2023 Sep 11;23:978. doi: 10.1186/s12913-023-09993-5 (PMC10494361; doi:10.1186/s12913-023-09993-5)
Supplement: Supplementary file 3 — Additional file 3. Socio-demographics questionnaire. [file 12913_2023_9993_MOESM3_ESM.pdf]

Additional file 3: Socio-demographics questionnaire

Patients

Please provide the following information about yourself. These data are provided with a coding number. After the evaluation, it will no longer be possible to draw conclusions about your person from the coding number.

1. Age: ______ years old

2. Gender:

☐ Female ☐ Male ☐ Diverse

3. Highest educational qualification

☐ No school-leaving qualification ☐ A-levels/Vocational baccalaureate/Matriculation

☐ Secondary school-leaving certificate ☐ Intermediate school-leaving certificate

☐ Vocational training ☐ Bachelor/Master craftsman/Business administrator

☐ Master's diploma/State examination ☐ PhD/Habilitation

☐ Other: ___________________________

4. Occupational status

☐ Unemployed ☐ Trainee, student

☐ Full-time employed ☐ Part-time employed

☐ Retired ☐ Other: ___________________________

5. Current or last occupation: ____

6. Membership of a support group for patients with knee osteoarthritis?

☐ Yes ☐ No

7. Date of diagnosis of knee arthrosis: ________________________ (month/year)

8. Date of surgery for total knee replacement

☐ Date of operation is not known

☐ Operation is planned for: ________________________ (month/year)

☐ Operation has been performed: ________________________ (month/year)

Doctors

Please provide the following information about yourself. These data are provided with a coding number. After the evaluation, it will no longer be possible to draw conclusions about your person from the coding number.

1. Age: ______ years old

2. Gender:

☐ Female ☐ Male ☐ Diverse

3. Highest educational qualification:

☐ State examination ☐ Medical doctor ☐ Habilitation

☐ other: ___________________________

4. Specialist training:

☐ Yes: ___________________________

☐ No

5. Field of activity

☐ General practice ☐ Specialist practice for orthopaedics

☐ Hospital ☐ Other: ___________________________

6. Work experience: ______ (years)

7. How often do you see patients with knee osteoarthritis in your practice?

_____ per week

8. Advanced training in evidence-based medicine and/or shared decision-making

☐ Yes: ____________________________________ ☐ No
