# Supplementary material for: Informed consent for total knee arthroplasty: exploration of patient`s information acquisition and decision-making processes—a qualitative study
Source: BMC Health Serv Res. 2023 Sep 11;23:978. doi: 10.1186/s12913-023-09993-5 (PMC10494361; doi:10.1186/s12913-023-09993-5)
Supplement: Supplementary file 4 — Additional file 4. Category scheme. [file 12913_2023_9993_MOESM4_ESM.pdf]

Additional file 4: Category scheme

Categories: Patients

Table 1: Patient categories

Category	Definition
**Information provision**	
Information provided at diagnosis	• Contains statements about information that patients received at the time of diagnosis of gonarthrosis regarding:<br>o the aetiology, course and treatment of the condition<br>o format of the information<br>o procedure for providing the information<br>o importance of the information<br>o source of information (general practitioner / specialist)
Information provided on treatment options	• Contains statements about information that patients received during the treatment of gonarthrosis regarding:<br>o treatment options<br>o format of the information<br>o the process of providing information<br>o importance of the information<br>o source of information (general practitioner / specialist)
Information provided regarding TKA	• Contains statements about information that patients received regarding knee replacement prior to informed consent:<br>o format of the information<br>o procedure for providing the information<br>o content regarding surgery<br>o content regarding anaesthesia<br>o importance of the information<br>o source of information (general practitioner / specialist)
Information provided in die informed consent process	• Contains statements about information that patients received during the informed consent process regarding:<br>o format of the information<br>o procedure for providing the information<br>o content regarding the operation<br>o content regarding anaesthesia<br>o importance of the information<br>o differences in the information provided

Table 1: continued

Category	Definition
**Information needs**	
Information needs at diagnosis	• Contains statements about information that patients would like to receive or expect regarding the aetiology, course and treatment of gonarthrosis:<br>○ questions for the treating doctor<br>○ missing information<br>○ importance of the desired information
Information needs regarding treatment options	• Contains statements about information that patients would like or expect to receive regarding various treatment options (excluding TKA):<br>○ questions for the treating doctor<br>○ missing information<br>○ importance of the desired information
Information needs regarding TKA	• Contains statements about information that patients would like to receive or expect to receive regarding TKA prior to the informed consent process:<br>○ questions for the treating doctor<br>○ missing information<br>○ importance of the desired information
Information needs in the informed consent process	• Contains statements about information that the patients would like to receive or expect to receive during the informed consent process:<br>○ questions for the treating doctor<br>○ missing information<br>○ importance of the desired information
**Information acquisition**	
Information acquisition	• Contains statements regarding patients' search for information on the aetiology, course and treatment (including TKA) of gonarthrosis:<br>○ sources of information, e.g. internet, friends, relatives, professionals<br>○ importance of the sources of information<br>○ evaluation of the information found / received<br>○ differences to the information provided by the doctors who treated the patient

Table 1: continued

Category	Definition
**Treatment course from diagnosis to TKA**	
Treatment course	• Contains statements describing the course of treatment from diagnosis to decision regarding knee replacement, including:<br>o treatments carried out / refused<br>o course of treatment over time<br>o doctor's visits due to the gonarthrosis
**Decision-making process**	
Decisions for / against treatment options (excluding TKA)	• Contains statements about the process of deciding for or against treatment options (excluding TKA) with regard to:<br>o factors influencing the decision<br>o persons involved in the decision<br>o involvement in the decision (Shared Decision-Making)<br>o course of the doctor-patient discussion<br>o expectations towards treating doctors<br>o value of the decision<br>o satisfaction with the decision-making process<br>o retrospective satisfaction with the decision
Decision for / against TKA	• Contains statements about the process of deciding for or against TKA regarding:<br>o factors influencing the decision<br>o persons involved in the decision<br>o involvement in the decision (Shared Decision-Making)<br>o course of the doctor-patient discussion<br>o expectations towards treating doctors<br>o value of the decision<br>o satisfaction with the decision-making process
**Role of the informed consent process and the informed consent form in the decision-making process**	
Procedure of the informed consent process	• Contains statements on the course of the informed consent process with regard to:<br>o time of the informed consent process<br>o time at which the informed consent form was received<br>o implementation<br>o expectations / personal needs
Decision-making processes within the informed consent process	• Contains statements regarding decisions made during the informed consent process with regard to:<br>o anaesthetic procedures<br>o alternative surgical options<br>o influence of the informed consent process / form

Categories: Outpatient doctors / clinician

Table 2: Outpatient doctors / clinicians' categories

Category	Definition
**Providing information from a doctor's perspective**	
Provision of information by outpatient doctors	• Contains statements by doctors regarding their own provision of information to patients with regard to:<br>○ content<br>○ format of the information<br>○ scope of the information<br>○ the process of providing information<br>○ dealing with patients who have already been informed<br>○ assessment of own abilities
Provision of information in the consultation and informed consent process from a clinical perspective	• Contains statements of clinicians regarding their own information transfer to patients in the consultation and informed consent process (anaesthesia/TKA) with regard to:<br>○ content<br>○ format of the information<br>○ scope of the information<br>○ procedure of information transfer<br>○ dealing with already informed patients<br>○ assessment of own abilities<br>○ inhibiting/encouraging characteristics of the informed consent process / the informed consent form used
**Decision-making processes from the doctor's perspective**	
Treatment options: Decision-making and inclusion of the patient perspective from the perspective of outpatient doctors.	• Contains statements from doctors regarding the decision-making process of patients in terms of:<br>○ involvement in decision making<br>○ inhibiting and facilitating factors for involvement in decision-making<br>○ assessment of own abilities
TKA: Decision-making and inclusion of the patient perspective in the informed consent process from the clinician's perspective.	• Contains statements from clinicians regarding the patient's decision-making process in terms of:<br>○ decision-making options<br>○ involvement in decision-making<br>○ inhibiting and facilitating factors for inclusion in decision-making<br>○ assessment of own abilities<br>• Importance of the informed consent process for the decision-making process
